# Cross‐Linked Versus Linear Hyaluronic Acid for Cartilage Repair in Rat Post‐Traumatic Osteoarthritis

**DOI:** 10.1002/jor.70213

**Published:** 2026-04-27

**Authors:** Tien‐Ching Lee, Chih‐Ming Kao, Yi‐Wun Lin, Yi‐Shan Lin, Yan‐Hsiung Wang, Hsuan‐Fu Chen, Yin‐Chih Fu

**Affiliations:** ^1^ Department of Orthopedics, Kaohsiung Medical University Hospital Kaohsiung Medical University Kaohsiung Taiwan; ^2^ Orthopaedic Research Center, College of Medicine, Kaohsiung Medical University Hospital Kaohsiung Medical University Kaohsiung Taiwan; ^3^ Regenerative Medicine and Cell Therapy Research Center Kaohsiung Medical University Kaohsiung Taiwan; ^4^ Department of Orthopedics, College of Medicine Kaohsiung Medical University Kaohsiung Taiwan; ^5^ School of Dentistry, College of Dental Medicine Kaohsiung Medical University Kaohsiung Taiwan; ^6^ Department of Medical Research Kaohsiung Medical University Hospital Kaohsiung Taiwan; ^7^ Superintendent Kaohsiung Show Chwan Memorial Hospital Kaohsiung Taiwan

**Keywords:** ACLT, cartilage repair, chondroprotection, cross‐linked hyaluronic acid, hyaluronic acid, post‐traumatic osteoarthritis, rat model, viscosupplementation

## Abstract

Post‐traumatic knee osteoarthritis develops following acute joint injuries and can progress rapidly, causing significant morbidity. Intra‐articular hyaluronic acid injections are a common treatment, but efficacy varies between formulations. We hypothesized that cross‐linked hyaluronic acid would provide superior chondroprotective and anti‐inflammatory effects compared to linear hyaluronic acid in a standardized post‐traumatic osteoarthritis model. Post‐traumatic osteoarthritis was induced via anterior cruciate ligament transection in 84 male Sprague‐Dawley rats assigned to four groups: Sham (*n* = 12), untreated osteoarthritis (*n* = 24), linear hyaluronic acid—Jetknee (*n* = 24), or cross‐linked hyaluronic acid—HYAJOINT Plus (*n* = 24). At each of four time points (Day 7, Week 4, Week 8, and Week 12 post‐surgery), three Sham and six rats per treatment group were sacrificed. Functional recovery was assessed by weight‐bearing distribution. Cartilage integrity was evaluated histologically using hematoxylin and eosin and Safranin O staining, scored with the Osteoarthritis Research Society International histopathological grading system. Immunohistochemical expression of type II collagen, type X collagen, matrix metalloproteinase‐13, and tumor necrosis factor‐alpha was analyzed. Both hyaluronic acid formulations significantly improved weight‐bearing compared to untreated controls. Cross‐linked hyaluronic acid demonstrated more sustained functional recovery and superior cartilage preservation with lower histological scores. Immunohistochemistry revealed reduced catabolic and inflammatory marker expression with higher type II collagen in the cross‐linked hyaluronic acid group. High molecular weight cross‐linked hyaluronic acid conferred stronger and longer‐lasting chondroprotective effects than linear hyaluronic acid, suggesting that structural optimization of hyaluronic acid formulations may confer more sustained chondroprotective effects in this preclinical model of post‐traumatic osteoarthritis.

## Graphical abstract

Cross‐linked HA outperforms linear HA in knee osteoarthritis.

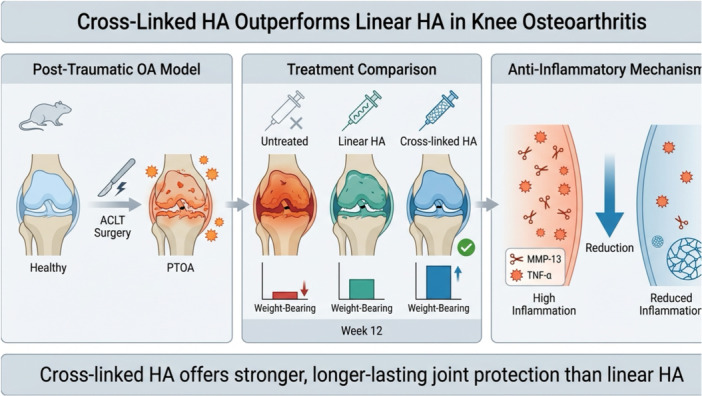

## 1 Introduction

Knee osteoarthritis (KOA) is the most common joint disease, characterized by progressive cartilage degeneration, chronic pain, and functional impairment [1, 2, 3]. Post‐traumatic osteoarthritis of the knee (PTOAK), a distinct subtype of KOA, develops following acute joint injuries such as intra‐articular fractures, meniscal tears, or ligament ruptures. The initial cartilage trauma, combined with subsequent pathobiological and biomechanical alterations, accelerates disease progression [4]. Additional risk factors for PTOAK development include advanced age, obesity, female gender, joint malalignment, and genetic predisposition [4, 5].

KOA significantly diminishes patients' health‐related quality of life and imposes substantial economic burden on healthcare systems worldwide through direct treatment costs and indirect productivity losses [6, 7]. Despite its growing prevalence, effective disease‐modifying pharmacological interventions to prevent or delay OA progression remain critically lacking, underscoring the urgent need for innovative therapeutic approaches [4, 8, 9, 10].

Hyaluronic acid (HA) is a high molecular weight, nonsulfated glycosaminoglycan that occurs naturally in synovial fluid and the extracellular matrix of articular cartilage. This viscoelastic biopolymer plays crucial roles in joint homeostasis, including synovial fluid lubrication, shock absorption, nutrient distribution, and maintenance of cartilage integrity [11, 12]. During OA progression, both the concentration and molecular weight of endogenous HA decrease significantly in the synovial fluid, compromising its rheological properties and protective functions [13]. Intra‐articular HA injections (viscosupplementation) have been utilized as a therapeutic intervention for osteoarthritis for several decades [14, 15, 16, 17]. The proposed mechanisms of action include restoration of synovial fluid viscoelasticity, reduction of pro‐inflammatory cytokines, inhibition of degradative enzymes, modulation of nociceptive pathways, and stimulation of endogenous HA production [11, 18]. Despite widespread clinical use, the therapeutic efficacy of HA injections continues to generate debate, with variable outcomes reported across clinical studies and meta‐analyses [17, 19, 20].

Recent advancements in HA technology, particularly molecular weight enhancement through cross‐linking and concentration optimization, have facilitated the development of next‐generation formulations with superior therapeutic profiles and extended intra‐articular residence time. These advanced formulations exhibit enhanced viscoelastic properties while simultaneously delivering multifaceted biological effects, including modulation of pro‐inflammatory cytokines, neutralization of reactive oxygen species, inhibition of nociceptive signaling, and preservation of chondrocyte function [21, 22]. Randomized controlled trials and systematic meta‐analyses have demonstrated that cross‐linked HA (CLHA) formulations with molecular weights exceeding 6 million Daltons achieve superior improvements in pain scores, functional outcomes, and patient satisfaction compared to lower molecular weight counterparts [23, 24, 25]. However, the precise molecular mechanisms governing differential tissue responses to various HA formulations remain incompletely characterized, and the temporal dynamics of cartilage matrix remodeling and inflammatory resolution following HA intervention require further elucidation.

While prior clinical evidence supports superior outcomes with higher molecular weight CLHA formulations, direct in vivo comparisons in a PTOA model—with temporal characterization of cartilage matrix and inflammatory responses—have not been reported. This study, therefore, investigates the comparative therapeutic efficacy of two distinct HA formulations in an anterior cruciate ligament transection (ACLT)‐induced rat model of PTOA [26, 27]. We compare standard linear HA (Jetknee) with high molecular weight CLHA (HYAJOINT Plus), examining their effects on cartilage structural integrity, inflammatory biomarker expression, and functional recovery. We hypothesize that the high molecular weight cross‐linked formulation will demonstrate superior chondroprotective properties and more substantial functional improvement compared to linear HA, potentially due to extended joint residence time and enhanced resistance to enzymatic degradation.

## 2 Methods

### 2.1 Experimental Design and Animal Model

A total of 84 male Sprague‐Dawley rats, each 11 weeks old and weighing between 405 and 450 g, were used in this study. All animals were obtained from BioLASCO Taiwan, a licensed laboratory animal supplier, and were specific pathogen‐free, wild‐type, and had not undergone any previous experimental procedures. Upon arrival, the rats were acclimatized for at least 1 week in the animal facility at Kaohsiung Medical University, where they were housed in individually ventilated cages with up to three rats per cage. The animal rooms were maintained at a temperature of 22°C ± 2°C and a relative humidity of 50%–60%, with a 12‐h light/dark cycle. Standard laboratory chow and tap water were provided ad libitum. To promote animal welfare and reduce stress, environmental enrichment such as nesting material (paper strips) and plastic tunnels was included in each cage. Cages were cleaned twice weekly, and all animals were monitored daily by trained staff for health and well‐being.

Following the acclimatization period, the rats were randomly assigned to one of four experimental groups using a random number table: (1) sham operation (Sham), (2) ACLT without treatment (OA), (3) ACLT followed by treatment with standard linear hyaluronic acid (OA/HA; Jetknee), and (4) ACLT followed by treatment with high molecular weight CLHA (OA/HP; HYAJOINT Plus). For each group, animals were sacrificed at four predetermined time points: Day 7, Week 4, Week 8, and Week 12 post‐surgery. At each time point, 3 rats from the Sham group and 6 rats from each of the other groups were included, resulting in a total of 12 rats in the Sham group and 24 rats in each of the OA, OA/HA, and OA/HP groups (Table 1). The sham group was included as a reference control to confirm baseline cartilage status under noninjured conditions and was not incorporated as a primary comparator in the inferential statistical analyses. The reduced sample size in this group was justified by the expected low biological variability and was consistent with the 3R principle of animal use reduction.

**Table 1:** Overview of experimental groups and animal numbers.

Group	Description	Day 7	Week 4	Week 8	Week 12	Total animals
Sham	Sham operation (no ACLT, no treatment)	3	3	3	3	12
OA	ACLT, no treatment	6	6	6	6	24
OA/HA	ACLT + linear HA (Jetknee)	6	6	6	6	24
OA/HP	ACLT + cross‐linked HA (HYAJOINT Plus)	6	6	6	6	24
Total		21	21	21	21	84

All surgical procedures and interventions were performed at the animal facility of Kaohsiung Medical University. The experimental unit in this study was an individual rat; all interventions, measurements, and analyses were conducted at the level of the single animal. All rats that completed the surgical procedure were included in the final analyses, and no animals or data points were excluded after randomization.

### 2.2 Blinding

During the allocation and conduct of the experiment, only the investigator performing the randomization and the therapeutic intervention was aware of group assignments. All surgeries, outcome assessments, and data analyses were performed by blinded investigators.

### 2.3 Surgical Procedure and Animal Care

All animals were fasted overnight prior to surgery. General anesthesia was induced via intramuscular injection of ketamine (87 mg/kg) and xylazine (13 mg/kg). Prophylactic antibiotics (Amikacin, 5 mg/kg, IM) were administered before the procedure. After skin disinfection with povidone‐iodine, a lateral parapatellar incision was made to expose the right knee joint. In the OA and treatment groups, the anterior cruciate ligament was transected using microscissors to induce post‐traumatic osteoarthritis. Sham‐operated animals underwent identical surgical exposure without ligament transection. The joint capsule and skin were closed with absorbable sutures.

Postoperative care included administration of analgesia (Ketolac, 1 mg/kg, IM) and a second dose of antibiotics (Amikacin, 5 mg/kg, IM). After surgery, animals were monitored under warming lamps for 30–60 min until full recovery from anesthesia before being returned to standard housing with unrestricted access to food and water. Wounds were inspected daily for signs of inflammation or infection.

To minimize pain, suffering, and distress, all animals received standardized perioperative analgesia and were provided with environmental enrichment in their home cages. Animals were observed at least once daily for clinical signs of pain, distress, infection, or abnormal behavior. Humane endpoints were predefined as follows: loss of more than 20% of baseline body weight, persistent severe pain or distress, wound infection or necrosis, or inability to eat or drink for over 24 h. Animals meeting any of these criteria were humanely euthanized by CO_2_ inhalation. No unexpected adverse events occurred during the study, and all animals that completed the surgical procedure were included in the final analysis.

### 2.4 Therapeutic Intervention

Two weeks after ACLT surgery, animals in the treatment groups received a single intra‐articular injection of either standard linear HA (Jetknee; OA/HA group) or high molecular weight CLHA (HYAJOINT Plus; OA/HP group). Each injection contained 20 μL of the respective formulation, delivered into the right knee joint cavity under sterile conditions.

### 2.5 Weight‐Bearing Distribution Analysis

**Figure 1:**
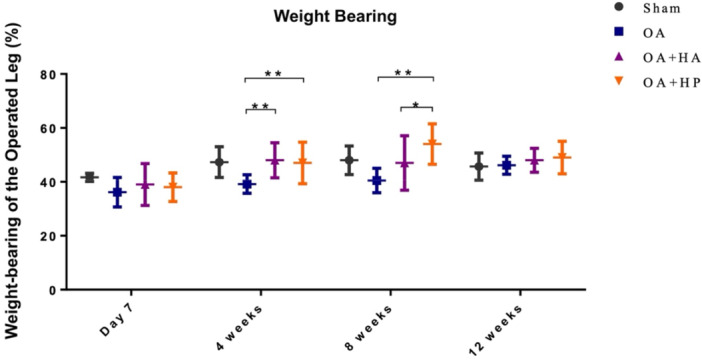
Weight‐bearing distribution of the operated hind limb following either sham surgery or anterior cruciate ligament transection (ACLT), with or without hyaluronic acid (HA) injection. Four groups were examined: (i) Ctrl (sham operation without ACLT), (ii) OA (ACLT with no injection), (iii) OA + HA (ACLT followed by standard linear HA injection, Jetknee), and (iv) OA + HP (ACLT followed by high molecular weight cross‐linked HA injection, HYAJOINT Plus). Weight‐bearing capacity was measured at designated intervals to assess functional recovery in each group. (*p < 0.05; **p < 0.01.)

Functional recovery was assessed using a dual‐channel weight average system (Singa Technology Corporation, Taipei, Taiwan). Measurements were taken at three time points: 1 week before ACLT surgery (baseline), 2 weeks after surgery (pretreatment), and immediately before sacrifice at the designated time points. For evaluation, rats were placed in a plexiglass chamber with each hind paw positioned on separate weight‐measuring platforms. A photograph of the measurement setup is provided in Supporting Information S3: Figure 1. Weight distribution was recorded for three 5‐s intervals, and the mean value was calculated. Weight‐bearing capacity was expressed as the percent difference between the operated and nonoperated hind limbs, serving as an indicator of joint discomfort and functional impairment.

### 2.6 Histological and Immunohistochemical Analysis

At the designated time points (Day 7, Week 4, Week 8, and Week 12 post‐surgery), animals were euthanized using carbon dioxide inhalation. Tibial plateau specimens were harvested, fixed in formalin, decalcified, and embedded in paraffin. Serial 5 μm sections were prepared for the following analyses.

#### 2.6.1 Histomorphological Evaluation

Sections were stained with hematoxylin and eosin (H&E) to assess overall cartilage architecture, cellular morphology, and tissue organization. Safranin O/Fast Green staining was performed to evaluate proteoglycan and glycosaminoglycan content within the cartilage matrix. Cartilage degeneration was quantified using the Osteoarthritis Research Society International (OARSI) histopathological scoring system, which assesses cartilage surface integrity, matrix depletion, chondrocyte clustering, subchondral bone changes, and synovial inflammation.

#### 2.6.2 Immunohistochemical Assessment

Expression of cartilage metabolism and inflammation markers was evaluated using the following primary antibodies:

1. Type II collagen (ARIGO, ARG20787; 1:1000 dilution): marker of healthy cartilage matrix.
2. Type X collagen (GeneTex, GTX37732; 1:200 dilution): indicator of chondrocyte hypertrophy and cartilage degeneration.
3. CD44 (GeneTex, GTX102111; 1:1000 dilution): hyaluronic acid receptor and mesenchymal stem cell marker.
4. Matrix metalloproteinase‐13 (MMP‐13) (GeneTex, GTX100665; 1:200 dilution): cartilage degradation enzyme.
5. Tumor necrosis factor‐alpha (TNF‐α) (Bioworld, BS1857; 1:500 dilution): pro‐inflammatory cytokine.

For immunohistochemical staining, sections were incubated with primary antibodies at room temperature for 16 h, followed by 30‐min incubation with secondary antibodies. Signal development was performed using appropriate chromogenic detection systems, followed by counterstaining and mounting.

### 2.7 Statistical Analysis

Sample size was determined based on a priori power analysis using the OARSI histopathological score as the primary outcome. Based on published data from comparable rat OA models reporting a required sample size of 6 animals per group to achieve 80% power at a significance level of *α* = 0.05 [28, 29], six rats per treatment group per time point were included, with three rats per time point for the sham control group. No additional buffer animals were allocated per group, given the low expected attrition rate under standard housing conditions. The sham group was not incorporated as a primary comparator in the inferential statistical analyses; details of the allocation rationale are provided in Section 2.1. Each time point was analyzed as an independent cross‐sectional comparison; no correction for multiplicity across time points was applied, as each animal contributed data at only one time point. This represents a potential limitation, and readers should interpret temporal trends with appropriate caution.

Since OARSI histopathological scores represent ordinal data with non‐normal distribution, nonparametric statistical methods were applied for all group comparisons [30, 31]. Intergroup differences at each time point were assessed using the Kruskal–Wallis one‐way analysis of variance. Post hoc pairwise comparisons were performed using Dunn's test, with the untreated OA group serving as the reference. Results were expressed as mean ± standard deviation. Statistical significance was defined as *p* < 0.05. All analyses were performed using Stata version 13 (StataCorp LLC, College Station, TX, USA).

### 2.8 AI Assistance Disclosure

During the preparation and revision of this manuscript, AI‐assisted tools were used to assist with English language editing and grammatical clarity. Specifically, Claude (Anthropic, Claude Sonnet 4.6, 2026) was used for language refinement and sentence restructuring. All scientific content, data interpretation, and conclusions were generated solely by the authors. All AI‐assisted edits were reviewed, verified, and approved by the authors, who accept full responsibility for the accuracy, integrity, and originality of the manuscript.

### 2.9 Ethical Statement and Protocol Registration

A detailed study protocol, including the research question, key design features, animal procedures, and analysis plan, was prepared prior to the initiation of the experiments and approved by the Institutional Animal Care and Use Committee of Kaohsiung Medical University (IACUC Approval No: 110020). This study was conducted in accordance with relevant guidelines and regulations. However, this protocol was not registered in a public repository or database. We have adhered to the ARRIVE guidelines and have supplied the ARRIVE Checklist.

## 3 Results

### 3.1 Weight‐Bearing Distribution Analysis

Weight distribution measurements revealed significant functional impairment following ACLT‐induced PTOAK (Supporting Information S1: Supplementary 1). Pretreatment assessment at 2 weeks post‐surgery showed significantly reduced weight‐bearing capacity in the operated limb across all ACLT groups (30%–35%) compared to the Ctrl group (39%–41%; *p* < 0.05). Following therapeutic intervention, both treatment groups demonstrated progressive improvement in weight‐bearing distribution (Figure 1).

The OA/HP group demonstrated more pronounced and sustained functional recovery, with weight‐bearing capacity increasing from 28% at Day 7 to 52% at Week 8, compared to the OA/HA group which improved from 30% at Day 7 to a peak of 46% at Week 12. In contrast, the untreated OA group maintained consistently impaired weight‐bearing throughout the observation period (range: 27%–35%), and both treatment groups exhibited significantly greater weight‐bearing capacity compared to the OA group at all post‐treatment time points (*p* < 0.05).

### 3.2 Histopathological Assessment

#### 3.2.1 OARSI Scoring of Cartilage Degeneration

**Figure 3:**
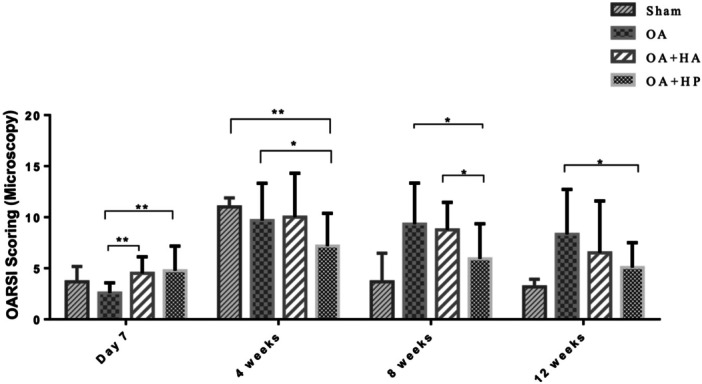
OARSI (Osteoarthritis Research Society International) scores for cartilage degeneration in four experimental groups: Control (Ctrl; sham‐operated), OA (ACLT‐induced without treatment), OA/HA (ACLT followed by a standard linear HA injection, Jetknee), and OA/HP (ACLT followed by a high molecular weight cross‐linked HA injection, HYAJOINT Plus) (*p < 0.05, **p < 0.01).

Histomorphological evaluation using H&E and Safranin O staining revealed progressive cartilage degeneration in the untreated OA group, while both treatment groups demonstrated chondroprotective effects (Figures 2 and 3, Supporting Information S2: Supplementary 2).

Figure 2Representative histological and immunohistochemical images of rat tibial plateau sections collected at (A) Day 7, (B) Day 28 (4 weeks), (C) Day 56 (8 weeks), and (D) Day 84 (12 weeks) post‐surgery. The four experimental conditions include: Control (Ctrl) sham‐operated, OA (ACLT‐induced with no treatment), OAHA (ACLT followed by standard linear HA injection, Jetknee), and OAHP (ACLT followed by high molecular weight cross‐linked HA injection, HYAJOINT Plus).

**Figure:**
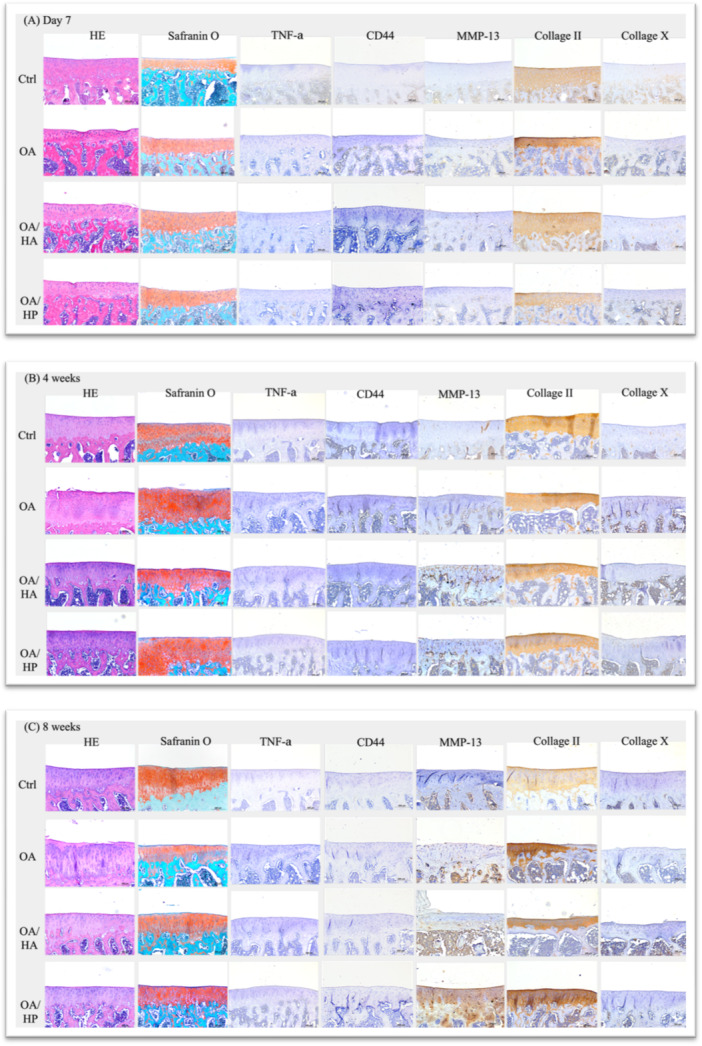

**Figure:**
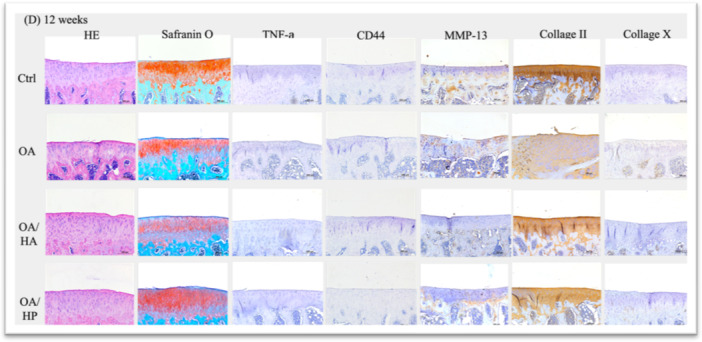

At Day 7 (pretreatment baseline), all ACLT groups exhibited early osteoarthritic changes with comparable OARSI scores (OA: 2.6 ± 0.17; OA/HA: 4.5 ± 0.54; OA/HP: 5.2 ± 0.87; Ctrl: 3.7 ± 0.41). Following treatment, the OA/HP group demonstrated significantly lower OARSI scores compared to the untreated OA group at all subsequent time points: Week 4 (6.7 ± 1.21 vs. 10.1 ± 1.43; *p* = 0.02), Week 8 (5.9 ± 1.41 vs. 9.3 ± 1.64; *p* = 0.03), and Week 12 (5.1 ± 0.99 vs. 8.3 ± 1.8; *p* = 0.03). While the OA/HA group also showed a trend toward cartilage preservation (Week 12: 6.5 ± 2.08), this did not reach statistical significance compared to the OA group at any time point.

### 3.3 Immunohistochemical Analysis of Cartilage Metabolism and Inflammatory Markers

#### 3.3.1 Type II Collagen (COL II) Expression

COL II expression followed a treatment‐dependent pattern throughout the study period (Figure 2). Both treatment groups maintained higher COL II levels than the untreated OA group from Week 4 onwards, with the OA/HP group consistently showing greater expression than the OA/HA group. Notably, at Week 8, the OA/HP group demonstrated the highest COL II expression among all groups, exceeding even the Ctrl group—suggesting enhanced maintenance of type II collagen expression; whether this reflects attenuated catabolism or anabolic stimulation warrants further investigation. By Week 12, both treatment groups showed comparable and elevated COL II expression relative to the OA group.

#### 3.3.2 Type X Collagen (COL X) Expression

COL X, a marker of chondrocyte hypertrophy and cartilage degeneration, was elevated in all ACLT groups at Day 7. Following treatment, both OA/HA and OA/HP groups demonstrated reduced COL X expression at Weeks 4, 8, and 12 compared to the untreated OA group, with comparable efficacy between the two formulations (Figure 2).

#### 3.3.3 Matrix Metalloproteinase‐13 (MMP‐13) Expression

MMP‐13 expression showed a dynamic pattern across time points (Figure 2). At Week 4, MMP‐13 levels were transiently elevated in the OA/HA group compared to the untreated OA group, potentially reflecting an early tissue remodeling response following linear HA injection. However, by Weeks 8 and 12, the OA/HP group demonstrated progressively lower MMP‐13 expression compared to both the OA and OA/HA groups, indicating more sustained suppression of cartilage catabolism with CLHA treatment.

#### 3.3.4 Tumor Necrosis Factor‐Alpha (TNF‐α) Expression

TNF‐α expression was undetectable in all groups at Day 7 and Week 4. At Week 8, minimal expression was observed exclusively in the untreated OA group. By Week 12, low‐level TNF‐α expression persisted in both the Ctrl and OA groups, while neither treatment group showed detectable expression, suggesting that both HA formulations effectively attenuated late‐stage joint inflammation (Figure 2).

#### 3.3.5 CD44 Expression

CD44 expression was minimal and inconsistent across groups and time points, precluding meaningful comparative analysis between treatment conditions (Figure 2).

## 4 Discussion

In this study, to our knowledge, we provide the first direct in vivo comparison of linear HA and CLHA formulations for PTOA. Using an ACLT rat model, we observed distinct advantages of a high molecular weight CLHA in functional and histopathological outcomes. Although both linear HA and CLHA facilitated significant improvements in weight‐bearing symmetry compared with untreated controls, CLHA consistently demonstrated more pronounced and sustained effects. This enhanced functional recovery was supported by histopathological assessment, which indicated better preservation of cartilage structure and reduced disease severity (reflected in OARSI scores) in CLHA‐treated joints. The increased expression of type II collagen in the CLHA group further supports the notion that cross‐linking, by increasing molecular weight and resistance to enzymatic degradation, may attenuate catabolic activity and support cartilage matrix maintenance in PTOA. Although these differences reached statistical significance, the absolute effect sizes were modest, and the biological relevance of these findings in the context of human disease remains to be established in future translational studies.

The therapeutic mechanisms by which HA exerts its effects likely involve a combination of mechanical and biological processes. Mechanically, both HA formulations improve synovial fluid viscoelasticity, thereby enhancing lubrication and shock absorption in the compromised joint. Biologically, HA modulates inflammatory pathways, chondrocyte metabolism, and extracellular matrix synthesis [11, 18]. Our immunohistochemical analysis revealed that both HA preparations attenuated catabolic and inflammatory markers. However, CLHA achieved stronger effects, notably in further reducing the expression of MMP‐13 at Week 12 and completely eliminating TNF‐α expression at Weeks 8 and 12. These findings align with emerging evidence that higher molecular weight formulations, afforded by cross‐linking, can extend intra‐articular residence time and optimize biological activity [21, 32, 33]. The durability of these viscoelastic and anti‐inflammatory effects likely underpins the sustained protection observed in the CLHA group, manifesting as improved weight‐bearing capacity and continued maintenance of cartilage integrity through 12 weeks post‐treatment.

HYAJOINT Plus—an HA formulation produced through biological fermentation and chemically cross‐linked with 1,4‐butanediol diglycidyl ether (BDDE) to achieve an exceptionally high molecular weight (> 15 million Daltons)—exhibited robust chondroprotective effects in our PTOA model. Reduced type X collagen expression in both linear and CLHA groups implies mitigation of chondrocyte hypertrophy. However, CLHA displayed additional benefits that appear critical to prolonging joint protection. BDDE‐CLHA has been widely employed in osteoarthritis therapies (e.g., Synvisc‐One, Durolane) owing to its favorable viscoelastic properties. Notably, recent prospective, double‐blinded randomized trials have shown that HYAJOINT Plus can outperform Synvisc‐One and Durolane in reducing pain, stiffness, and patient‐reported discomfort [23, 34]. While these clinical data pertain mostly to primary KOA, our findings expand upon these observations by demonstrating more sustained chondroprotective and anti‐inflammatory effects of this particular CLHA formulation in a preclinical post‐traumatic model. This suggests that PTOA may benefit especially from more prolonged residence time and mechanical stabilization afforded by cross‐linked, high molecular weight HA formulations.

Beyond pharmacological and intra‐articular interventions, effective OA management typically involves a multifaceted approach that addresses both mechanical and biological contributors to disease progression. According to current guidelines, lifestyle modifications such as weight management and structured exercise programs (including low‐impact aerobic exercises and strength training) are fundamental components of care, as they can reduce excessive joint loading and promote better musculoskeletal function [35, 36]. Physical therapy further helps maintain or restore joint range of motion, muscle strength, and neuromuscular control, thereby alleviating pain and improving functional capacity [37, 38]. In parallel, emerging therapies—such as platelet‐rich plasma [39, 40], stem cell [41, 42], exosome injections [43], and novel biologic agents aimed at modulating inflammatory or catabolic pathways—have shown promise in early‐stage clinical evaluations [44, 45]. When integrated appropriately, these modalities may create a synergistic effect, potentially enhancing the clinical benefits conferred by HA‐based treatments [46, 47, 48]. By embedding HA interventions, particularly high molecular weight cross‐linked formulations, into a broader therapeutic strategy that includes exercise, weight control, and selective use of biologics, clinicians may achieve more robust and sustained outcomes for patients across varied OA etiologies and severity levels.

Despite these encouraging findings, several limitations warrant consideration when interpreting the results of this study. First, although the ACLT‐induced rat model is well‐established for PTOA research, it may not fully capture the complexity of human OA. In particular, the accelerated disease progression commonly observed in rodent models can amplify treatment effects that might be subtler in clinical settings. Second, this study employed a single intra‐articular injection protocol (20 μL), and future clinical applications may require optimization of both injection regimens and volume. Third, the relatively short follow‐up period of 12 weeks may not sufficiently reveal differences in the long‐term efficacy and durability of linear versus CLHA formulations; further complicating this issue is the likelihood that HA degradation kinetics in rats differ from those in humans. Fourth, weight‐bearing distribution, while an established functional outcome in rodent OA models, demonstrated notable inter‐animal variability, and the observed between‐group differences were modest in absolute terms. The sensitivity of this measure to detect biologically meaningful differences may therefore be limited. More comprehensive functional assessments—including gait analysis and behavioral pain scores—would provide a fuller characterization of treatment‐related functional recovery. In addition, the focus on select histopathological and immunohistochemical markers (type II collagen, type X collagen, CD44, MMP‐13, TNF‐α) may not fully capture the breadth of molecular pathways involved in differential HA responses. Notably, CD44 expression was minimal and inconsistent across groups and time points, precluding meaningful comparative analysis; this may reflect limitations in antibody sensitivity under the tissue processing conditions used, or suggest that CD44‐mediated HA signaling plays a more context‐dependent role in PTOA than previously postulated. Finally, this study specifically evaluated a PTOA model induced by mechanical injury, potentially limiting the generalizability of the results to other OA etiologies (e.g., age‐related, metabolic, or inflammatory). Future investigations incorporating alternative models, extended follow‐up periods, a broader molecular marker panel, and comprehensive functional assessments will be crucial for refining our understanding of how different HA formulations may optimize clinical management of OA in various patient populations.

## 5 Conclusion

This study demonstrates that both linear and CLHA formulations effectively support functional recovery and modulate the joint microenvironment in a rat PTOAK model, evidenced by favorable shifts in cartilage metabolic markers and reduced inflammatory mediator expression. High molecular weight CLHA provided enhanced and sustained chondroprotective effects compared to linear HA, likely attributable to its greater molecular weight and resistance to enzymatic degradation, suggesting that structural optimization of HA formulations may more effectively modulate cartilage catabolism and attenuate joint inflammation in preclinical PTOA models.

Future studies should focus on elucidating the molecular mechanisms underlying these differential effects, establishing optimal dosing and injection timing protocols for various OA phenotypes, and evaluating the translational potential of CLHA in prospective human clinical trials.

## Author Contributions

Conceptualization: Yin‐Chih Fu. Methodology: Tien‐Ching Lee, Chih‐Ming Kao, and Yan‐Hsiung Wang. Software: Yi‐Wun Lin. Validation: Yi‐Shan Lin and Yan‐Hsiung Wang. Formal analysis: Yi‐Wun Lin. Investigation: Tien‐Ching Lee, Chih‐Ming Kao, and Hsuan‐Fu Chen. Resources: Yin‐Chih Fu. Data curation: Yan‐Hsiung Wang. Writing – original draft: Tien‐Ching Lee. Writing – review and editing: Yin‐Chih Fu. Visualization: Yi‐Wun Lin. Supervision: Yin‐Chih Fu and Yan‐Hsiung Wang. Project administration: Tien‐Ching Lee and Yin‐Chih Fu. Funding acquisition: Yin‐Chih Fu. All authors have read and agreed to the published version of the manuscript.

## Ethics Statement

A detailed study protocol, including the research question, key design features, animal procedures, and analysis plan, was prepared prior to the initiation of the experiments and approved by the Institutional Animal Care and Use Committee of Kaohsiung Medical University (IACUC Approval No: 110020).

## Conflicts of Interest

The authors declare no conflicts of interest.

## Supporting information

Supporting File 1

Supporting File 2

Supporting File 3

## Data Availability

The data that support the findings of this study are partially available as Supporting Material. Additional data are available from the corresponding author upon reasonable request.
