# Supplementary figures and images for: Cross‐Linked Versus Linear Hyaluronic Acid for Cartilage Repair in Rat Post‐Traumatic Osteoarthritis
Source: J Orthop Res. 2026 Apr 27;44:e70213. doi: 10.1002/jor.70213 (PMC13112325; doi:10.1002/jor.70213)

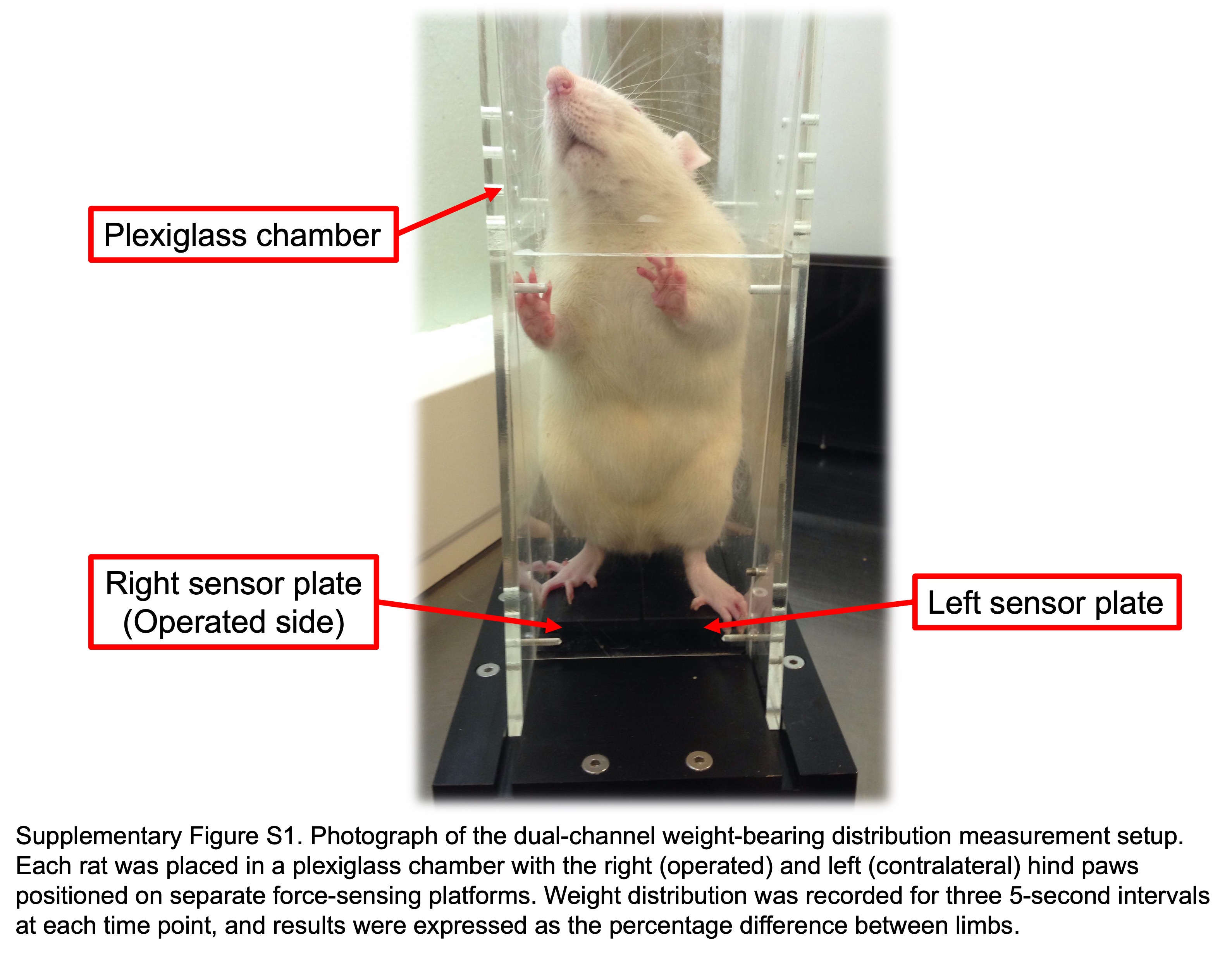

Supplement: Supplementary file 3 — Supporting File 3 [file JOR-44-0-s003.jpg]
